# Diffusion Tensor Imaging in an Infant Undergoing Functional Hemispherectomy: A Surgical Aid

**DOI:** 10.7759/cureus.1697

**Published:** 2017-09-18

**Authors:** Allen L Ho, Arjun V Pendharkar, Eric S Sussman, May Casazza, Gerald A Grant

**Affiliations:** 1 Department of Neurosurgery, Stanford University School of Medicine; 2 Division of Pediatric Neurosurgery, Lucile Packard Children's Hospital

**Keywords:** diffusion tensor imaging, epilepsy, epilepsy surgery, hemispherectomy, otoharra syndrome, white matter tracts, pediatric neurosurgery

## Abstract

Hemispherectomy is a highly effective treatment option for children with severe, unilateral, medically refractory epilepsy. Many patients undergoing hemispherectomy are younger patients with dysmorphic brains, making accomplishing a complete disconnection challenging due to anatomic distortion, even with the aid of intraoperative navigation. Diffusion tensor imaging (DTI) has been proposed as a valuable imaging adjunct perioperatively to help guide surgeons intraoperatively, as well as for post-surgical evaluation and confirmation of complete hemispheric disconnection. We present a case of an infant with Otoharra syndrome and hemimegencephaly who underwent a functional hemispherectomy for treatment of severe, refractory seizures. We demonstrate how DTI was utilized both pre-, intra-, and postoperatively to help plan, guide, and confirm surgical disconnection. The application of exquisite DTI for this child led to her being seizure-free, which is a life-changing event with long-lasting benefits and will become even more critical as we now perform these disconnection procedures with a more minimally invasive approach.

## Introduction

Hemispherectomy is an effective treatment option for children with severe, unilateral, medically refractory epilepsy. Despite significant surgical modifications in technique to allow for greater efficacy and less morbidity [1-2], surgical failure is still a common occurrence necessitating repeat surgery. Rates of seizure freedom in recently published series range from 60-80% [2-4]. Many patients undergoing hemispherectomy are younger patients with dysmorphic brains, making accomplishing a complete disconnection challenging due to anatomic distortion, even with the aid of intraoperative navigation. The most common areas of incomplete disconnection are, in order, the frontal-basal cortex, corpus callosum, and insular cortex [5]. Diffusion tensor imaging (DTI) has been proposed as a valuable imaging adjunct perioperatively to help guide surgeons intraoperatively, as well as for post-surgical evaluation and confirmation of complete hemispheric disconnection.  

We present a case of an infant with Otoharra syndrome and hemimegencephaly who underwent functional hemispherectomy for treatment of severe, refractory seizures. We demonstrate how DTI was utilized both pre-, intra-, and postoperatively to help plan, guide, and confirm surgical disconnection.

## Case presentation

**Figure 1:**
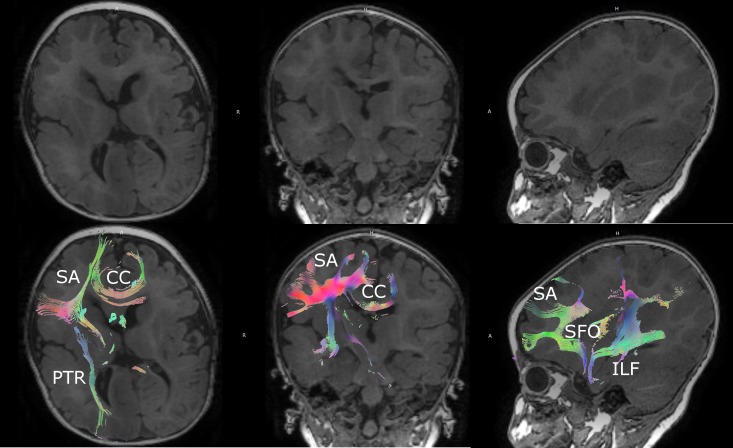
Preoperative magnetic resonance imaging with diffusion tensor imaging Preoperative imaging demonstrating right hemimegencephaly and manual seeding of right hemispheric white matter tracts for preoperative planning and intraoperative neuronavigation. CC: corpus callosum; PTR: posterior thalamic radiations; SA: short association fibers; SFO: superior fronto-occipital fasciculus; ILF: inferior longitudinal fasciculus

The patient is a seven-month-old female born with right-sided hemimegencephaly and Otoharra syndrome leading to intractable seizures (Figure 1). She spent most of her life in and out of hospitals for seizure management and was on five antiepileptic medications (AEDs), including clobazam, phenytoin, lacosamide, phenobarbital, topiramate, and a ketogenic diet at the time of surgery. Her seizure semiology was eye deviation towards the left, left-sided rhythmic arm movements, and lip-smacking that at times progressed to full generalized tonic-clonic seizures. Previous video electroencephalograms (EEGs) demonstrated right hemisphere hemihypsarrhythmia with a burst suppression pattern during sleep and right hemispheric seizures. The patient was transferred to our facility for further seizure management and consideration of epilepsy surgery. Video EEG at time of admission confirmed focal subclinical status epilepticus despite an aggressive AED regimen.

A multidisciplinary pediatric epilepsy team conference was held with the patient’s family and the decision was made to proceed with surgery due to the frequency and severity of seizures, despite multiple antiepileptic medications and the unilateral onset due to hemimegalencephaly. The patient was taken to the operating room for a functional hemispherectomy. The procedure was staged in two parts over the course of six days due to the patient’s young age and concern for the development of coagulopathy due to significant blood loss. Preoperatively, a full magnetic resonance imaging (MRI) scan with DTI was obtained for planning purposes that demonstrated a severely distorted right hemispheric anatomy due to right hemimegencephaly (Figure 1, Video 1). White matter tracts visualized with DTI aided to help plan disconnections preoperatively. This scan was then utilized intraoperatively with the DTI sequences fused with the AxiEM™ neuronagivation system (Medtronic, Inc., Louisville, CO) to delineate the anatomy and aid with the disconnection surgery by providing real-time localization of target white matter tracts. A functional hemispherectomy was completed with frontobasal and posterior disconnections all the way to the falx and down to the ventricles, as well as complete corpus callosotomy and temporal lobectomy. Factor VIIA was infused during the case for hemostasis, which was helpful due to the risk for a coagulopathy intraoperatively in this young child due to diffuse intravascular coagulation.  An external ventricular catheter was left in the right ventricle. 

**Video 1:** Preoperative three-dimensional diffusion tensor imaging Three-dimensional reconstruction of right hemispheric white matter tracts manually seeded from preoperative diffusion tensor imaging.

**Figure 2:**
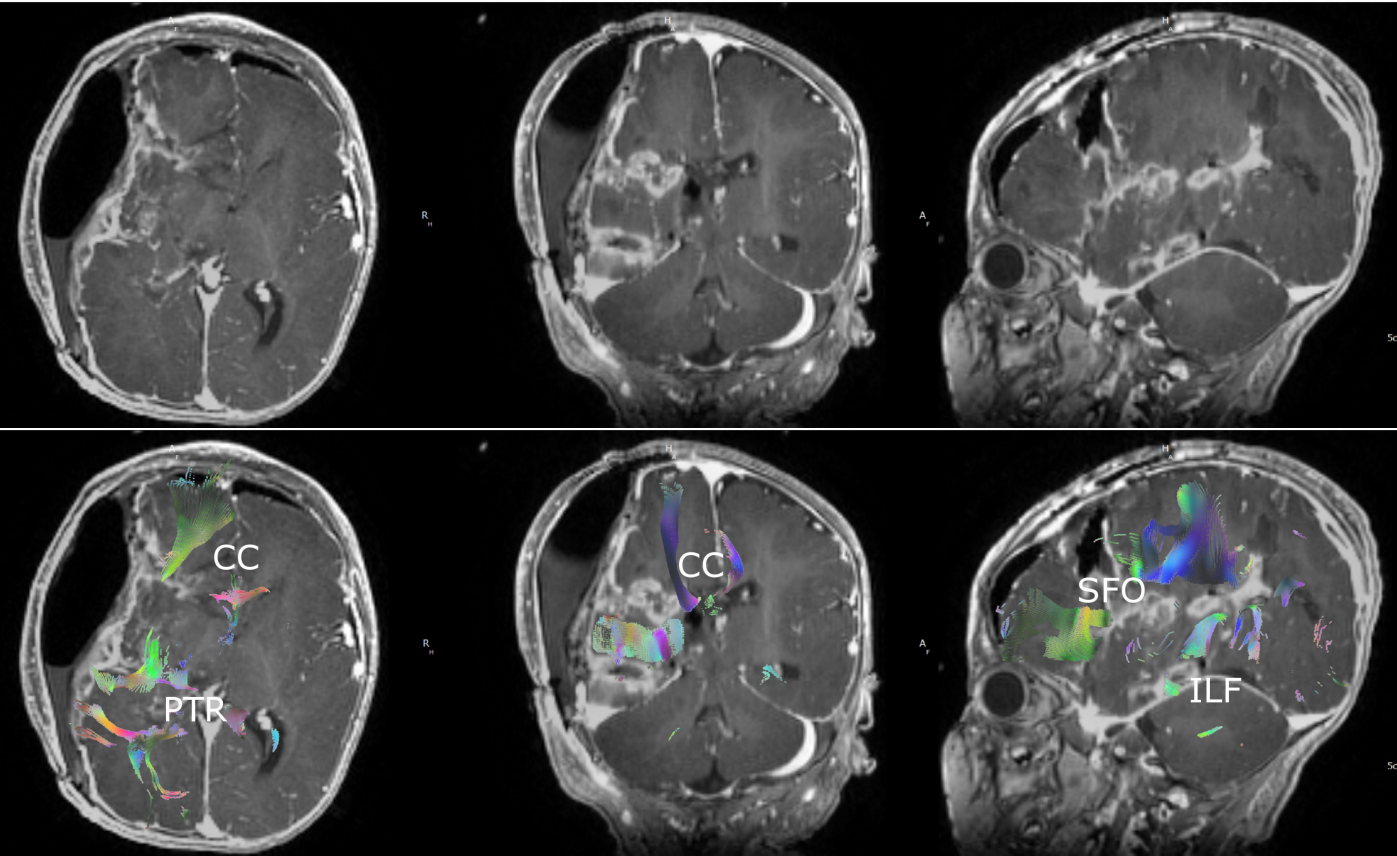
Postoperative magnetic resonance imaging (MRI) with diffusion tensor imaging Postoperative MRI demonstrating expected postoperative changes and manual seeding of right hemispheric white matter tracts demonstrating disconnections consistent with complete functional hemispherectomy. CC: corpus callosum; PTR: posterior thalamic radiations; SFO: superior fronto-occipital fasciculus; ILF: inferior longitudinal fasciculus

Postoperatively, the patient did well and was extubated on postoperative day (POD) 2 and did not have any further clinical seizures during her hospitalization. Postoperative MRI obtained on POD 1 demonstrated expected postoperative changes with evidence of completion of functional hemispherectomy on DTI with interruptions of targeted white matter tracts throughout the right hemisphere (Figure 2, Video 2). The patient was initially plegic on the left side following surgery but gradually recovered movement in both the left upper and lower extremities. Patient was discharged on POD 14 to inpatient rehabilitation in stable condition. At her three-month follow-up, patient had not had any further clinical seizures and her left-sided weakness continued to improve.

**Video 2:** Postoperative three-dimensional diffusion tensor imaging Three-dimensional reconstruction of right hemispheric white matter tracts manually seeded from postoperative diffusion tensor imaging demonstrating complete disconnection of hemisphere status-post right hemispherectomy.

## Discussion

Ohtahara syndrome, also known as early infantile epileptic encephalopathy, is considered the earliest of the age-dependent epileptic encephalopathies. There is a poor neurologic outcome with a mortality rate of 50% in infancy. The majority of cases are associated with a structural brain malformation. Because of the high morbidity and mortality associated this disorder, hemispherectomy and aggressive resection of focal dysplasias have been proposed as the primary treatment. Limited studies have shown that in select patients with Ohtahara syndrome, the outcome was better in those treated surgically than those treated medically [5-6]. Focal resections involving temporal and frontal lobes are usual procedures in children with cortical malformations being the most common underlying pathology. Hemispherectomy or multilobar procedures are, however, more commonly performed in children younger than four years.

Hemispherectomy has evolved over time to become an invasive but efficacious treatment for severe, medically refractory, hemispheric epilepsy from a wide variety of acquired, developmental, and progressive etiologies [6]. The surgery itself is a complicated procedure of many steps that are sometimes staged, as in this case, and is particularly challenging in younger infants and patients with poorly developed hemispheric anatomy [7]. In this patient with a right hemimegencephaly, anatomic distortion made tissue planes and normally reliable gyral and sulcal surfaces more difficult to appreciate. The patient was also an infant, so blood loss considerations and the risk of development of severe coagulopathy were also significant concerns. Intraoperative adjuncts, such as intraoperative neuronavigation with DTI that allowed for less surgical misadventure and decreased operative time, were critical.

Overall, seizure freedom rates following successful hemispherectomy are quite favorable. A recent longitudinal study of a large cohort of patients reported seizure freedom rates of 78% at six months, 76% at one year, and 71% at two years [2]. Another meta-analysis of seizure freedom rates following hemispherectomy reported an overall 61% seizure freedom rate in 169 patients. The most common reason for failure after hemispherectomy has been due to incomplete disconnection. This has been reported in 12.9% - 54.5% of patients. This is also supported by the fact that reoperation in patients with suboptimal seizure control can lead to improvement in seizure control [8]. Indeed, in our patient, seizures continued to occur in the interval between her two surgeries before complete disconnection had been achieved. DTI allows for the identification of residual white matter connections that serve as a conduit for continued seizure propagation and can provide additional functional data over conventional anatomic imaging. It has been widely utilized for planning and intraoperative purposes for intra-axial brain tumor resection, but the utilization in epilepsy surgeries has been limited until recently. In two recent studies, DTI was utilized to detect residual white matter connections after initial hemispherectomy. Kiehna, et al. identified eight patients with recurrent seizures following initial hemispherectomy. In all patients, DTI imaging demonstrated residual connecting white matter tracts, primarily across the rostrum/genu of the corpus callosum. Following reoperation, five of eight of these patients achieved complete seizure freedom [9]. In another recent study, Kim, et al. utilized intraoperative MRI with DTI to identify residual tracts. In over a third of cases, intraoperative MRI with DTI helped identify incomplete disconnection, prompting further surgery and confirmatory MRI with DTI. This methodology helped achieve complete disconnection in 94% of patients confirmed on postoperative MRI with DTI and 91% seizure freedom rates in patients with median follow-up of 1.7 years [10]. Thus, utilization of DTI imaging following hemispherectomy, as illustrated in our present case, can be an invaluable tool for confirming complete disconnection of target white matter tracts intraoperatively and postoperatively.

## Conclusions

As higher resolution and more functional imaging modalities, such as DTI, become more ubiquitous and integrated for intraoperative imaging and image-guided navigation, it is important that they are applied in a functional manner to make the goals of surgery more attainable. We present an illustrative case of how perioperative MRI with DTI can be an essential surgical aid in an anatomically complex and technically challenging hemispherectomy surgery. As more and more centers adopt this technology with the ability to recreate these highly valuable and intuitive images intraoperatively, we are hopeful that adoption of DTI imaging for epilepsy disconnection surgeries will help improve outcomes and decrease seizure rates.
